# Immunoglobulin E induces VEGF production in mast cells and potentiates their pro-tumorigenic actions through a Fyn kinase-dependent mechanism

**DOI:** 10.1186/1756-8722-6-56

**Published:** 2013-08-02

**Authors:** Guillermina Yanek Jiménez-Andrade, Alfredo Ibarra-Sánchez, Diana González, Mónica Lamas, Claudia González-Espinosa

**Affiliations:** 1 Departamento de Farmacobiología, Cinvestav, IPN, Sede Sur, Calzada de los Tenorios 235, Col. Granjas Coapa, Tlalpan CP 14330, Mexico City, Mexico; 2 Department of Pediatrics, University of California, San Francisco, San Francisco, CA 94143-0519, USA

**Keywords:** Immunoglobulin E, Mast cells, VEGF, Fyn tyrosine kinase, Melanoma, Angiogenesis, Inflammation

## Abstract

**Background:**

High concentrations of plasmatic IgE have been related to distinct systemic inflammatory conditions that frequently predispose individuals to hypersensitivity reactions. Although effects of IgE have been suggested to relay on the low-intensity activation of distinct effector elements of the immune system, such as mast cells (MC), experimental evidence on the role of IgE-induced production of inflammatory mediators on specific pathologies is scarce. MC are an important component in tumor microenvironment where they seem to secrete a number of immunomodulatory and angiogenic mediators, such as the Vascular Endothelial Growth Factor (VEGF) by not well-described mechanisms. In this work, we investigated the effect of monomeric IgE (in the absence of antigen) on the production of VEGF in MC, analyzed if monomeric IgE could exacerbate the pro-tumorigenic properties of that cell type and characterized some of the molecular mechanisms behind the effects of IgE on VEGF production and tumor growth.

**Methods:**

For *in vitro* studies, murine bone marrow-derived mast cells (BMMCs) were used. Pharmacological inhibitors and phosphorylation of key elements controlling VEGF secretion and protein translation were used to characterize the mechanism of VEGF production triggered by IgE.

*In vivo*, the effect of a single i.v. administration of monomeric IgE on B16 melanoma tumor weight, intratumoral blood vessel formation and tumor-associated MC was assessed in four groups of mice: MC-proficient (WT), MC-deficient (Wsh), Wsh reconstituted with MC derived from WT mice (Wsh Rec WT) and Wsh reconstituted with MC derived from Fyn −/− mice (Wsh Rec Fyn −/−).

**Results:**

Monomeric IgE induced VEGF secretion through a Fyn kinase-dependent mechanism and modulated *de novo* protein synthesis modifying the activity of the translational regulator 4E-BP1 in BMMCs. *In vivo*, monomeric IgE increased melanoma tumor growth, peritumoral MC and blood vessel numbers in WT but not in Wsh mice. The positive effects of IgE on melanoma tumor growth were reproduced after reconstitution of Wsh mice with WT but not with Fyn −/− BMMCs.

**Conclusion:**

Our data suggest that monomeric IgE, in the absence of antigen, induces VEGF production in MC and *in vivo* contributes to melanoma tumor growth through a Fyn kinase-dependent mechanism.

## Background

High concentrations of plasmatic IgE are related to a number of systemic inflammatory conditions [1] and recent evidence has lead to propose that inflammation predisposes individuals to certain types of cancer [2]. Some data indicate that underlying infections and inflammatory responses are linked from 15 to 20% of all deaths due to cancer worldwide [3].

Mast cells (MCs) are central players in allergic reactions and also constitute a well documented component of tumor microenvironment [4,5]. The pleiotropic actions of this cell type are associated with its remarkable capacity of synthesis and secretion of diverse lipid mediators after IgE/antigen stimulation. Proteases, cytokines, chemokines and growth factors (i.e. Tumor Necrosis Factor, TNF; Interleukin (IL)-1,2,3,4,6, and 13, and the Vascular Endothelial Growth Factor, VEGF) secreted by MC have been proposed to modulate tissue remodeling, immune response and angiogenesis [5,6].

The best characterized stimulus for MC activation is the crosslinking of the high affinity IgE receptor (FcϵRI), which occurs after the interaction of IgE bound to the receptor with specific antigens [4]. After FcϵRI triggering, two Src-family kinases (Lyn and Fyn) are activated to initiate different intracellular events leading to cytokine production [7,8]. It has been well documented that Lyn phosphorylates key sites on ITAM motifs of the receptor, initiating signaling and activating mechanisms of negative control of signaling [9], whereas Fyn kinase has a positive regulatory role for the activation of PI3K and leukotriene production [10].

The initial binding of monomeric IgE to the receptor was considered for long time as a “sensitization” event for late antigen-dependent stimulation. Recent *in vitro* studies have shown, however, that non-specific IgE, in the absence of antigen, is able to modify MC secretory profile in distinct cell preparations and cell lines. Those changes, produced by IgEs with no relevant recognition for specific antigens, have been hypothesized to be relevant to the initiation of local inflammatory reactions, especially in humans with high levels of circulating IgE, like atopic individuals [1,11]. However, to date, the effect of monomeric IgE on the production of angiogenic factors such as VEGF and its consequences on inflammation-related angiogenesis is not well-described.

MC activation is closely related to tumor growth [12,13]. Specifically, in human and murine melanoma biopsies, increased numbers of MC correlate with a high microvascular density in tumors and poor prognosis [14]. In addition, a strong significant correlation has been found between the number of VEGF-positive peritumoral MC, microvessel density and aggressive melanoma [15]. The mechanisms of MC activation that could contribute to the secretion of pro-angiogenic factors have not been fully described.

The objective of this work was threefold 1) to test if monomeric IgE (in the absence of antigen) could induce the production of VEGF in MC *in vitro*; 2) to analyze if monomeric IgE could exacerbate the pro-tumorigenic properties of this cell type *in vivo*; and 3) to investigate some of the molecular mechanisms underlying the effects of IgE on VEGF production and tumor growth.

## Results

### Monomeric IgE induces VEGF secretion in MC through a mechanism that requires *de novo* synthesis, tetanus toxin-sensitive VAMPs and the activity of Fyn kinase

**Figure 1:**
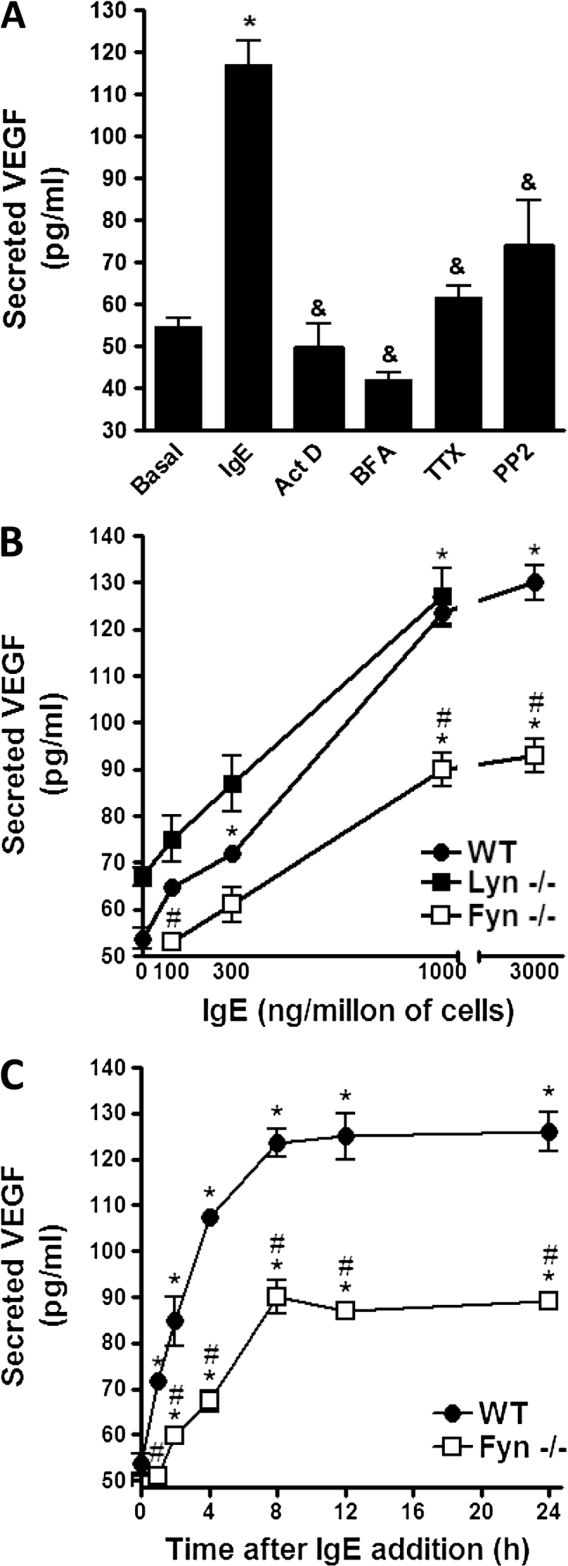
**Monomeric IgE induces secretion of VEGF in BMMCs through a mechanism that requires Fyn. (A)** Pharmacological characterization of IgE-induced VEGF secretion in MC. Two million BMMCs were pre-incubated with vehicle, Actinomicyn D (Act D; 5 μg/ml), Brefeldin A (BFA; 5μg/ml), Tetanus toxin (TTx; 10 ng/ml) and PP2 (10 μM) for 15 min in BMMC media. Then, cells were stimulated with 1000 ng of IgE for 8 h at 37°C. VEGF in cell supernatants was quantified by ELISA. **(B)** Role of different Src family kinases on IgE-induced VEGF secretion in MC. Two million BMMCs derived from WT, Fyn −/− and Lyn −/− mice were incubated with different amounts of IgE at 37°C for eight hours. Supernatants were collected and VEGF was determined by ELISA. **(C)** Time-course of VEGF secretion after the addition of IgE. Two million BMMCs were incubated in cell culture media containing 1000 ng/million cells of IgE at 37°C and supernatants were collected at different times after stimulation. VEGF was determined by ELISA. All results are shown as the mean ± SEM (n = 3-12). *, P < 0.05 compared with basal or time 0, &, P < 0.05 compared with IgE treatment, #, P < 0.05 compared to WT. One way ANOVA post hoc Student-Newman-Keuls **(A)**. Two way ANOVA post hoc Student-Newman-Keuls **(B** and **C)**.

Production of angiogenic factors by MC has been shown to occur few hours after different stimuli, such as hypoxia, antigen or PMA [16,17]. To investigate if monomeric IgE in the absence of any antigen could induce VEGF secretion in this cell type, two million BMMCs were incubated with a monoclonal anti-DNP IgE (1000 ng/ml) for eight hours at 37°C in BMMC media. Supernatants were then collected and the amount of secreted VEGF was determined by ELISA. The addition of IgE to MC induced a significant secretion of VEGF (53.77 ± 2.15 pg/ml in basal conditions vs 117.16 ± 5.45 pg/ml on IgE-stimulated cells; Figure 1A). To gain insight on the mechanism involved in IgE-induced VEGF production, cells were pre-treated with different pharmacological inhibitors 15 minutes before the addition of IgE and secreted VEGF was measured. VEGF secretion was sensitive to actinomycin D (ActD) and brefeldin A (BFA), indicating that *de novo* transcription and transport from endoplasmic reticulum to the Golgi apparatus [18] was needed for IgE effects to occur. VEGF production was also affected by tetanus toxin (TTx), which inhibits secretion mediated by toxin-sensitive VAMPs (VAMP-1 and −2) [19], and PP2, that inhibits Src family kinases (Figure 1A).

Two main Src family kinases modulate mediator secretion from MCs. Lyn and Fyn kinases have been shown to be involved in early signaling after FcϵRI crosslinking, leading to the activation of downstream pathways regulating pro-inflammatory cytokine production [7]. In order to test if one of them could be involved in IgE-induced VEGF secretion in BMMC, cells from WT, Lyn −/− and Fyn −/− mice were generated and stimulated with different concentrations of monomeric IgE (Figure 1B). WT BMMCs reached maximal VEGF secretion after the incubation with 1000 ng/million cells while BMMCS generated from Lyn −/− mice did not show an important difference when compared to WT cells. However, BMMCs derived from Fyn −/− mice showed an important defect on IgE-induced VEGF production, since the maximal amount of secreted VEGF in Fyn −/− cells was significantly lower than in WT cells (Figure 1B). We then characterized the time course of VEGF secretion stimulated by IgE in WT and Fyn −/− cells. VEGF in cell supernatants was detectable as early as two hours after stimulation and the maximal amount was reached after eight to twelve hours of stimulation in both cell types. However, Fyn −/− BMMCs did not secrete as much VEGF as WT cells even at longer incubation times (Figure 1C).

### Monomeric IgE in the absence of antigen induces intracellular VEGF accumulation in a Fyn kinase-dependent fashion

**Figure 2:**
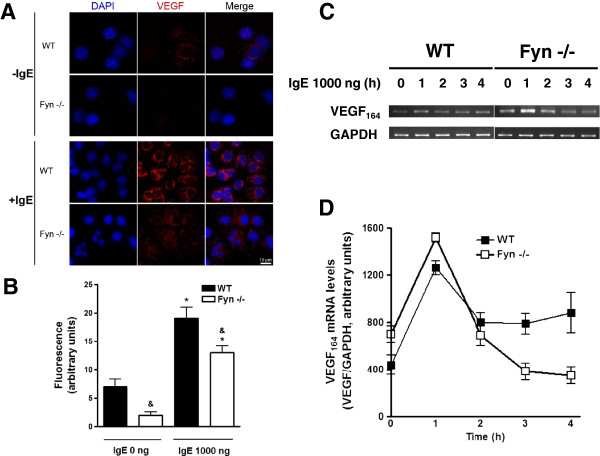
**IgE-induced VEGF protein but not mRNA accumulation is affected in Fyn −/− BMMCs. (A** and **B)** Intracellular VEGF accumulation after IgE treatment of MC. Two million WT and Fyn −/− BMMCs were stimulated with 1000 ng of IgE for eight h at 37°C. Cells were collected in a cytospin and slides were incubated in the presence of an anti-VEGF antibody (red) and DAPI (blue). Samples were processed to detect intracellular VEGF by confocal microscopy. A representative picture obtained from at least three independent experiments is shown. Scale bar = 10 μm. Mean fluorescence intensity values of VEGF signal obtained from experiments shown in A (n = 3) are represented in B. *, P < 0.0001 compared with IgE 0 ng; &, P < 0.001 compared with WT. Two way ANOVA post hoc Student-Newman-Keuls. **(C** and **D)** VEGF mRNA expression in IgE-stimulated MC. Total mRNA was purified from WT or Fyn −/− BMMCs treated with 1000 ng IgE and VEGF_164_ mRNA was amplified by RT-PCR. GAPDH amplification is shown as a control. Densitometric quantification of VEGF_164_ mRNA (n = 2) is shown in **D**.

In order to investigate the role of Fyn kinase in the IgE-dependent VEGF synthesis, BMMCs derived from WT or Fyn −/− mice were incubated at 37°C in the presence of vehicle or IgE (1000 ng/ml) for 8 hours. Cells were collected using a cytospin centrifuge and processed to detect VEGF by fluorescent immunostaining. Figures 2A and B show that low VEGF positive signal was observed in WT BMMCs and IgE addition increased intracellular VEGF. In contrast, VEGF immunoreactivity was significantly lower in Fyn −/− BMMCs in both basal and IgE-stimulated conditions.

In order to investigate if the absence of Fyn could lead to alterations on IgE-dependent VEGF mRNA synthesis, we analyzed VEGF mRNA content by RT-PCR in WT and Fyn −/− BMMCs after IgE stimulation. Optimal conditions for amplification of VEGF and GAPDH (control) were experimentally determined in our cell system (see Methods section). VEGF_164_ mRNA, the predominant isoform in different cell systems including MC [13], was increased in WT BMMCs after IgE addition and the same effect was observed in Fyn −/− cells. Interestingly, Fyn −/− BMMCs showed slightly higher amounts of VEGF mRNA than WT BMMCs in the absence of any stimulus and also one hour after IgE addition (Figure 2C-D). Together, these results strongly suggested that Fyn kinase could be involved on the translational control of VEGF protein production.

### IgE alters MC protein translation through the Fyn-dependent 4E-BP1 dephosphorylation

**Figure 3:**
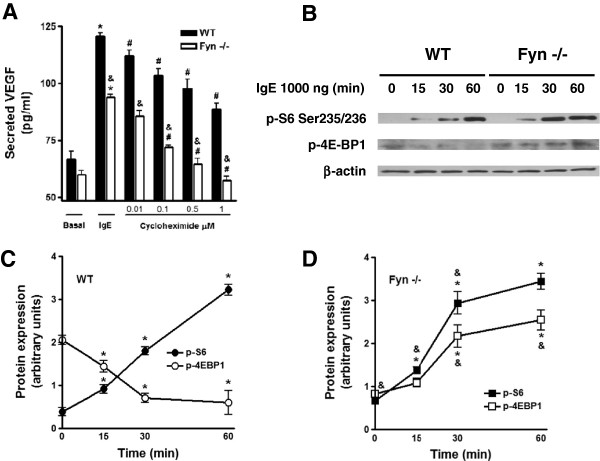
**Fyn is involved in IgE-induced VEGF protein translation and in IRES-dependent translation. (A)** IgE-induced VEGF synthesis is sensitive to cicloheximide. Two million BMMCs were pre-incubated with cycloheximide (CHX) for 15 min in cell culture media at 37°C. Then, cells were stimulated with 1000 ng of IgE for eight hours. VEGF was determined in cell supernatants. Results are shown as the mean ± SEM (n = 3). *, P < 0.05 compared with basal, #, P < 0.05 compared with IgE-treated cells. &, P < 0.05 compared with WT cells. Two way ANOVA post hoc Student-Newman-Keuls. **(B)** Effect of IgE on the activation of translational regulators. Two million BMMCs were stimulated with 1000 ng of IgE for 0, 15, 30 and 60 min in cell culture media. Western blot analysis of S6 and 4E-BP1 protein phosphorylation induced by IgE in MC. β-actin was used as a loading control. Samples were normalized to their respective β-actin loading control and data are expressed as arbitrary units. **(C)** Quantification of S6 and 4E-BP1 phosphorylation in WT BMMCs. **(D)** Quantification of S6 and 4E-BP1 phosphorylation in Fyn −/− BMMCs. Mean ± SEM (n = 3). *, P < 0.05 compared with 0 ng of IgE; &, P < 0.05 compared with WT. Two way ANOVA post hoc Student-Newman-Keuls.

To test the hypothesis that IgE-induced VEGF protein translation could be defective in the absence of Fyn, we evaluated whether VEGF synthesis in Fyn −/− cells could be more sensitive to the protein synthesis inhibitor cycloheximide (CHX) than in WT BMMCs. We found that CXH pre-treatment provoked a decrease on VEGF production in both cell types, but Fyn−/− BMMCs showed an increased sensitivity to this inhibitor (Figure 3A). The main difference was detected at concentrations of 1 μM of CHX, where normalized VEGF production was inhibited by 26.53% of the maximal release in WT cells, compared to 52.49% in Fyn −/− BMMCs.

It has been shown that VEGF synthesis under different stressful conditions (such as hypoxia) depends on the rapid modification of protein translation machinery [20]. In those circumstances, the normal 5′cap-dependent translation is stopped and internal ribosome entry sites (IRES)-dependent translation proceeds on VEGF mRNA [20]. Those adjustments on the translational apparatus can be followed by changes on the phosphorylation of the ribosomal protein S6 (S6) and the dephosphorylation of the eukaryotic initiation factor 4E binding protein 1 (4E-BP1) [21,22]. To investigate if IgE could induce a stressful condition similar to hypoxia in MC leading to IRES-dependent protein translation, we analyzed the phosphorylation levels of pS6 and 4E-BP1 in WT and Fyn −/− BMMCs stimulated with IgE.

Figure 3B shows that IgE addition to BMMCs induce a rapid phosphorylation of S6 and dephosphorylation of 4E-BP1 in WT cells. In contrast IgE-dependent, dephosphorylation of 4E-BP1 was not detected in the absence of Fyn. Quantitative analysis of western blots performed for Figure 3B is presented on Figure 3C-D. Optical density values of each specific band were normalized with those obtained from actin in the same membrane (see Material and Methods section). Results show that IgE is able to induce changes on the translational machinery of MC, promoting IRES-dependent translation in a Fyn-dependent manner.

### Non-specific monomeric IgE increases B16 melanoma tumor growth in a MC-dependent fashion

In order to evaluate the effect of IgE on the secretion of VEGF in MC and the role of Fyn kinase in that process *in vivo*, we took advantage of the well-recognized role of MC and VEGF on the development of B16 melanoma tumor [15,23]. A number of experiments have shown that MC have a positive effect on murine melanoma angiogenesis and tumor growth [23,24]. For instance, MC-deficient mice (Wv strain) are unable to generate angiogenesis of B16 melanoma tumors and the reconstitution of those animals with WT BMMCs leads to the restoration of tumor development [23]. The number of VEGF positive MC located in the peritumoral area has been found to correlate with increased angiogenesis and poor prognosis of melanoma [15].

We started defining the amount of IgE that could be administered in mice to analyze its effect on melanoma tumor growth. We decided to utilize a dose of IgE that could give a measurable parameter of MC and basophil activation. Passive anaphylactic reactions have been shown to depend on MC activity when initiated with an specific IgE and triggered with the corresponding antigen [25,26]. Exogenous IgE occupies free FcϵRI receptors on immune cells and those can be crosslinked administrating the antigen to which IgE was synthesized [27].

**Figure 4:**
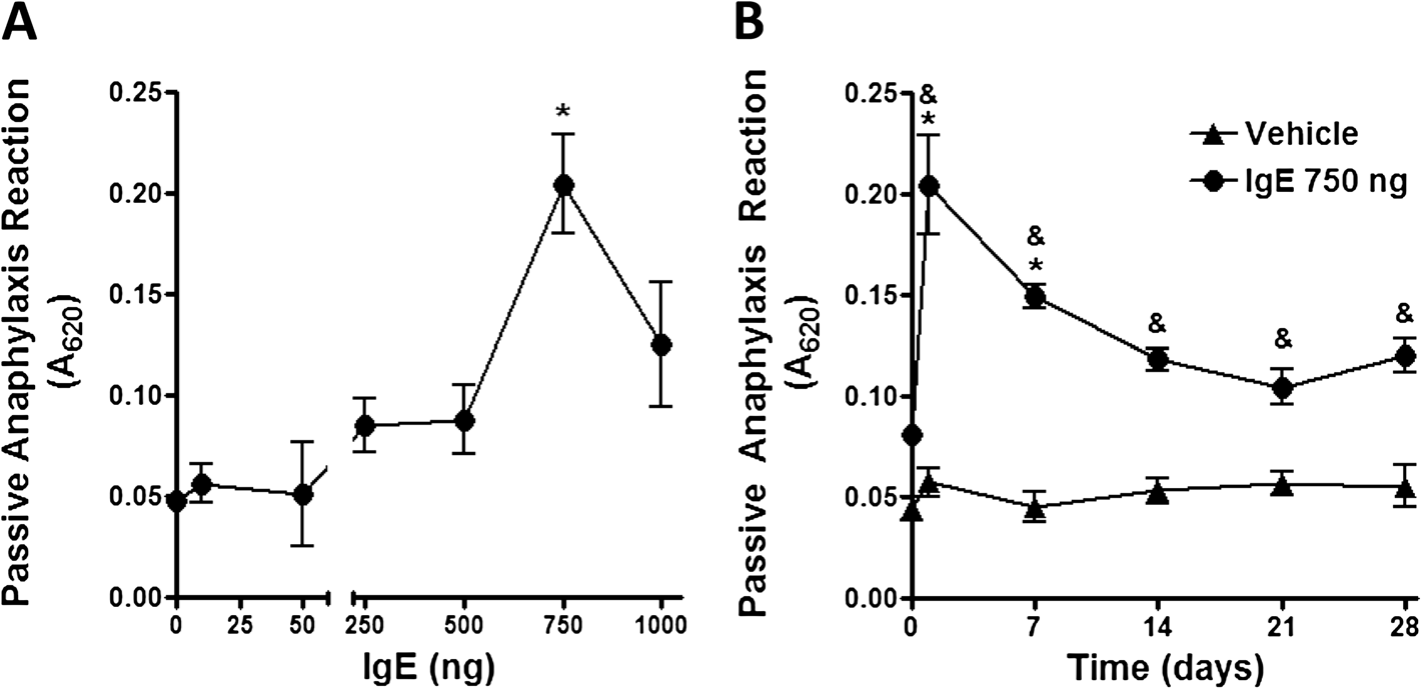
**IgE causes sustained increase on specific anaphylactic reaction. (A)** Effect of IgE on anaphylactic reaction. C57BL/6J mice were i.v. administered with discrete amounts of monomeric IgE directed against DNP-HSA. Twenty four hours later, antigen (100 μg) in 100 μl of Evans blue dye was i.v. injected and twenty minutes later, a passive cutaneous anaphylaxis assay was performed. A_620_ represents the absorbance at 620 nm of the extravasation dye in the limbs. Results are shown as the mean ± SEM (n = 4-10). **(B)** Time-course of specific passive cutaneous anaphylaxis after a single administration of IgE. C57BL/6J mice were i.v. administered with monomeric IgE directed against DNP-HSA (750 ng), antigen was i.v. injected in Evans blue dye at days 1, 7, 14, 21 and 28 after sensitization with IgE. Twenty minutes later, a passive cutaneous anaphylaxis assay was performed. Results are shown as the mean ± SEM (n = 7-10). *, P < 0.05 versus 0 ng of IgE; &, P < 0.05 versus vehicle.

We determined the concentration of IgE needed for maximal activation of passive anaphylactic reaction in C57BL/6J mice. Monomeric IgE directed against DNP-HSA was intravenously (i.v.) injected to mice and twenty four hours later, antigen was i.v. injected in the presence of Evans blue dye. Twenty minutes after antigen administration, limbs were removed and dye extravasation was measured. As can be observed in Figure 4, the optimal IgE dose to obtain maximal anaphylactic response was 750 ng per mouse and the effect of IgE was long-lasting, since antigen-specific anaphylactic reaction was observed in the limbs even four weeks after a single IgE administration.

**Figure 5:**
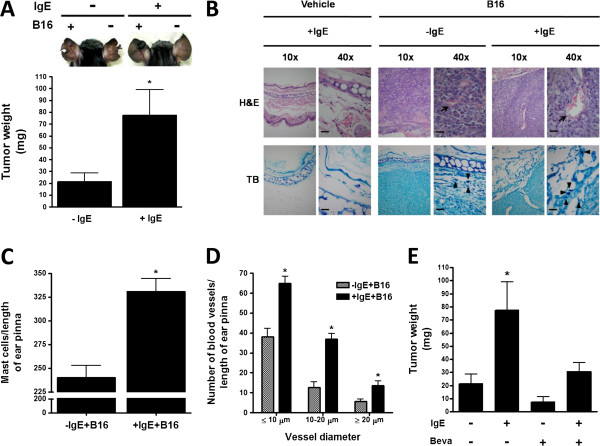
**IgE causes increased melanoma tumor growth. (A)** Effect of IgE on B16 melanoma tumor weight in C57BL/6J mice. Animals were treated with a single i.v. administration of saline (−IgE) or monoclonal anti-DNP IgE (750 ng/mouse; +IgE). Twenty four hours later, mice were s.c. inoculated with B16 melanoma cells (+B16) in one ear pinna and tumor weight was determined after four weeks of inoculation. Upper panel, a representative picture of vehicle or IgE-treated mice and vehicle or B16 cells-treated ear pinna. Lower panel, quantification of tumor weight. Results are shown as the mean ± SEM (n = 11-12). *, P < 0.05 versus + IgE mice. **(B)** Histological analysis of tissue biopsies. Ear pinna sections (2.5 μm) were stained with H&E (upper pictures) and TB (lower pictures). Blood vessels are indicated by arrows and MC by arrowheads. Photos are representative images from distinct mice (n = 2-4). Scale bar = 20 μm. **(C)** Quantification of MC by TB staining. Data are expressed as mean ± SEM from two sections per mouse, (n = 2-4). *, P < 0.05 versus –IgE + BL6. **(D)** quantification of blood vessels per ear pinna in tissue sections. Mean ± SEM (n = 3). *, P < 0.05 versus –IgE + BL6. **(E)** Effect of Bevacizumab on the IgE-dependent effects on melanoma tumor growth. Mice were treated with Bevacizumab (Beva; 10 mg/kg; s.c.) biweekly, starting 24 h after inoculation of melanoma cells. Ear pinna were removed and tumors were measured as in panel A. Data are expressed as the mean ± SEM (n = 4-11). *, P < 0.05 versus –IgE + BL6.

To evaluate the effect of IgE on melanoma tumor growth, C57BL6/J (WT) mice were treated with a single i.v. administration of saline or monomeric IgE (750 ng/mouse) and twenty four hours later, mice were subcutaneously (s.c.) inoculated with B16 melanoma cells in one ear pinna and generated tumors were removed after four weeks. Tumors obtained from IgE-treated mice (+IgE) were larger than those obtained from saline-treated mice (−IgE) (Figure 5A). Histopathologic analysis of tumor sections utilizing hematoxilin-eosin (H&E) and toluidine blue (TB) stains showed that those obtained from IgE-treated mice presented significantly higher numbers of blood vessels inside the tumor and MC in the peritumoral area (Figure 5B-D). The role of VEGF on melanoma tumor growth was verified in our system by the use of the neutralizing anti-VEGF antibody Bevacizumab (Beva; Figure 5E). As expected, administration of Beva (10 mg/kg) diminished tumor size. Remarkably, the effect of IgE on tumor growth was prevented by the administration of that agent.

**Figure 6:**
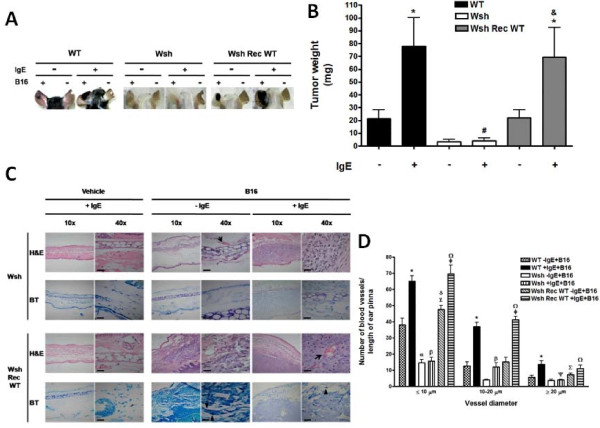
**IgE improves the pro-tumorigenic properties of MC. (A** and **B)** Participation of MC on IgE-induced melanoma tumor growth. C57BL6/J (WT), Wsh and Wsh Rec WT mice were treated with a single i.v. administration of saline (−IgE) or monoclonal anti-DNP IgE (750 ng/mouse; +IgE). Twenty four hours later, mice were s.c. inoculated with B16 melanoma cells (+B16) in one ear pinna and tumor weight was determined four weeks after inoculation. Representative pictures of tumors from WT, Wsh and Wsh Rec WT mice after four weeks of inoculation are shown in A. Mean tumor weight of WT, Wsh and Wsh Rec WT mice. Mean ± SEM (n = 7-12). *, P < 0.05 versus IgE-treated mice; #, P < 0.05 versus WT IgE-treated mice; &, P < 0.05 versus Wsh IgE-treated mice. **(C)** Histological analysis of tissue biopsies. Ear pinna sections (2.5 μm) were stained with H&E and TB. Blood vessels are indicated by arrows and MC by arrowheads. Pictures are representative images (n = 2-3). Scale bar = 20 μm. **(D)** Quantification of blood vessels in ear pinna from treated animals. Mean ± SEM (n = 3). *, P < 0.05 versus WT-IgE + B16; α, P < 0.001 versus WT-IgE + B16; β, P < 0.001 versus WT + IgE + B16; λ, P < 0.05 versus WT-IgE + B16; δ, P < 0.001 versus Wsh-IgE + B16; ø, P < 0.001 versus Wsh Rec WT-IgE + B16; Ω, P < 0.001 versus Wsh + IgE + B16; Ψ, P < 0.01 versus WT + IgE + B16; ∑, P < 0.05 versus Wsh-IgE + B16.

When B16 melanoma cells were inoculated in MC-deficient mice (Wsh), the size of the tumors after 28 days was significantly lower than the observed in WT mice (Figure 6A-B). As expected, normal tumor size was recovered after the reconstitution of Wsh animals with BMMCs derived from WT mice (Wsh Rec WT). Interestingly, monomeric IgE importantly increased the size of melanoma tumors on WT and Wsh Rec WT mice but this effect was not observed in Wsh animals treated with IgE. The effect of IgE on melanoma tumor growth positively correlated with the capacity of IgE to induce anaphylactic reactions, since Evans blue extravasation in Wsh Rec WT mice was similar to the observed in C57BL6/J mice under the same treatment (data not shown).

Histological analysis of biopsies of Wsh and Wsh Rec WT-derived tumors, in the presence or absence of IgE, was performed by staining slides with H&E and TB dyes. The number of blood vessels and peritumoral MC was significantly higher in Wsh Rec WT than in Wsh mice (Figure 6 C-D).

### Fyn kinase in MC is required for IgE-induced melanoma tumor growth

**Figure 7:**
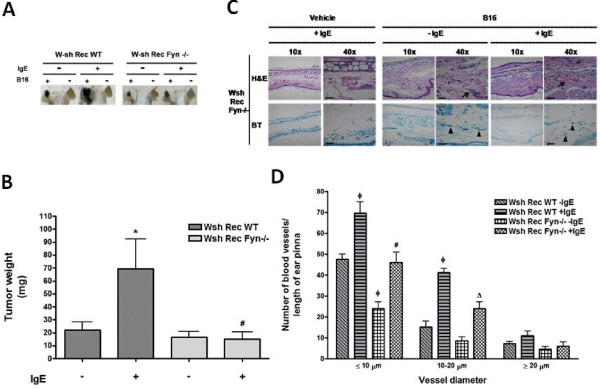
**Fyn kinase facilitates MC-mediated melanoma tumor growth. (A** and **B)** Role of Fyn kinase in MC on IgE-induced melanoma tumor growth. Wsh Rec WT and Wsh Rec Fyn −/− mice were treated with a single i.v. administration of saline (−IgE) or monoclonal anti-DNP IgE (750 ng/mouse; +IgE). Twenty four hours later, mice were s.c. inoculated with B16 melanoma cells (+B16) in one ear pinna and tumor weight was determined after four weeks of inoculation. Representative pictures of tumors from Wsh Rec WT and Wsh Rec Fyn−/− mice after four weeks of inoculation are shown in A. Results are shown as the mean tumor weight from Wsh Rec WT and Wsh Rec Fyn−/− mice. Mean ± SEM (n = 3-11). *, P < 0.05 compared with IgE-treated mice. #, P < 0.05 compared with Wsh Rec WT IgE-treated mice. **(C)** Histological analysis of tissue biopsies. Ear pinna sections (2.5 μm) were stained with H&E and TB. Blood vessels are indicated by arrows and MC by arrowheads. Pictures are representative images (n = 3-4). Scale bar = 20 μm. **(D)** Quantification of blood vessels in ear pinna from treated mice. Mean ± SEM (n = 3). #, P < 0.05 versus Wsh Rec WT + IgE + B16; ø, P < 0.001 versus Wsh Rec WT-IgE + B16; Δ, P < 0.01 versus Wsh Rec WT + IgE + B16.

To investigate if Fyn kinase in MC could be involved in IgE-induced melanoma growth and angiogenesis, Wsh mice were reconstituted with BMMCs from Fyn −/− mice (Wsh Rec Fyn−/−). IgE-dependent tumor growth was significantly impaired in mice reconstituted with Fyn−/− BMMCs compared to mice reconstituted with WT cells (Figure 7A-B). A decreased number of blood vessels and MC were observed in tumor biopsies obtained from Wsh Rec Fyn−/− mice compared to those obtained from Wsh Rec WT animals (Figure 7C).

## Discussion

A possible link between inflammation and cancer was first proposed in the nineteenth century, based on observations that tumors often arose at sites of chronic inflammation and that inflammatory cells were present in biopsies from tumors [3]. Since then, the critical relationship between inflammation and cancer development has been under investigation [2]. Controversial results have been obtained from studies that analyze the relationship between allergic inflammation and cancer. It has been found that allergies increase the risk of bladder cancer, lymphoma, myeloma, and prostate cancer. However, a decreased risk has been reported among allergies and glioma, colorectal cancer, cancer of the larynx, non-Hodgkin lymphoma, cancer of the esophagus, oral cancer, pancreatic cancer, stomach cancer, and uterine body cancer [28].

In this paper we show that monomeric IgE (with irrelevant capacity of tumor recognition and in the absence of antigen) induces VEGF production in isolated MC through a Fyn kinase-dependent mechanism, and this exacerbates pro-tumorigenic properties of this particular cell type *in vivo*.

Our results show, for the first time, that nonspecific monomeric IgE promotes the synthesis of pro-angiogenic factors modulating the protein translation system in MC*.* Although high concentrations of monomeric IgE have been shown to activate MC leading to the production of inflammatory mediators [29-31] we were able to detect VEGF production in lower concentrations of IgE, indicating that secretion of this pro-angiogenic factor could occur in the absence of extensive FcϵRI aggregation.

Pharmacological characterization of IgE-induced VEGF production indicates that this factor is transported from ER-to-Golgi and is secreted in vesicles decorated with TTx-sensitive VAMPs. This mechanism resembles the reported pathway of hypoxia-induced VEGF secretion in BMMCs [16]. The observed pharmacological profile contrasts with that leading to pre-formed mediators exocytosis, such as β-hexosaminidase [16], which requires TTx-insensitive VAMPS and is resistant to brefeldin treatment. Our data suggest that monomeric IgE and hypoxia could share some common effectors that participate in cytokine production, since both stimuli induce VEGF release without extensive MC degranulation tested by β-hexosaminidase activity in the supernatant of IgE-treated cells (data not shown).

The modifications of the translational apparatus associated to VEGF production were analyzed and it was found that monomeric IgE induced dephosphorylation of 4E-BP1 in WT MC, suggesting an increase on IRES-dependent protein translation. In contrast, 4E-BP1 dephosphorylation was not observable in the absence of Fyn, where even an increase on 4E-BP1 phosphorylation was detected after IgE treatment. Our data show for the first time that monomeric IgE causes changes on the translational machinery of MC, favoring IRES-dependent protein translation, in a Fyn dependent-manner. Since the IRES-dependent mechanism of protein translation occurs preferentially during hypoxic conditions [20], it is possible to speculate that IgE could induce some signaling pathways activated also by hypoxia. Current experiments in our laboratory have been designed to determine if monomeric IgE activates similar pathways to hypoxia in MC.

When IgE was administered *in vivo*, an increased size of melanoma tumors was observed in WT and in Wsh-reconstituted mice. To our knowledge, this is the first report showing that non-specific IgE promotes melanoma tumor growth and angiogenesis in a MC-dependent fashion. Loading MC with circulating IgE has been proposed to rapidly occur due to the formation of specific cellular structures in MC that are able to penetrate the endothelial cell layer of blood vessels [32]. On the other hand, binding of IgE to the FcϵRI receptor has been associated with an increase of MC cytokine synthesis and MC survival [29,30], so, the observed increase on tumor growth could be due to the secretion of MC-derived inflammatory mediators or MC survival stimulated by IgE.

MC-derived VEGF seems to contribute to active angiogenic processes observed in some tumors [15]. The effect of IgE on tumor growth was sensitive to bevacizumab but the obtained value was not statistically significant, suggesting the participation of VEGF but also other compounds on IgE actions. It has been shown that other mediators such as tryptase released by mast cells play an important role in neovascularization [33] and a close relationship has been demonstrated between tryptase-positive MC and tumor vascularity in melanoma [14].

The positive influence of MC on tumor growth has been demonstrated utilizing MC-deficient mice (Kit^Wv/Wv^) [23]. Our results with Wsh mice, a model used to study B16 melanoma growth and metastasis [34], confirm those findings and extend the observation to include the participation of monomeric IgE as an important stimulus for MC activation and Fyn kinase as a central molecule controlling pro-tumorigenic actions of MC.

Fyn kinase is an important effector of FcϵRI signaling system. Its activation after IgE/Antigen stimulation of MC leads to degranulation, leukotriene synthesis and selective cytokine expression [10,35]. However, its role on monomeric IgE-mediated effects on MC seems to be discrete, i.e. MC survival after IgE treatment requires Lyn but not Fyn activation [36] and IgE-induced adhesion to fibronectin was also shown to be independent of Fyn [37]. Here we show for the first time that IgE-induced VEGF synthesis requires Fyn activity, describing a non-recognized role of this kinase on IgE-induced cytokine production.

Administration of tumor-specific mouse monoclonal IgE antibodies prevent the development of mammary adenocarcinoma [38] and inhibit colorectal carcinoma growth [39]. Those and other studies have suggested that IgE could exert a protective role against tumors [40]. In our study, a non-specific IgE was injected to mice and our data support the idea that IgE is able to induce pro-angiogenic factors that favour tumor growth. Differences among our results and those obtained with anti-tumor IgEs could be explained by the fact that SPE-7 clone has been shown to be able to induce increased cytokine synthesis in distinct MC preparations *in vitro*[31]. Since it has been proposed that some allergic patients might produce cytokinergic IgE’s [11], our data are relevant to the study of physiological conditions where high plasmatic concentrations of non-specific IgEs are reached, such as atopy [1].

## Conclusions

**Figure 8:**
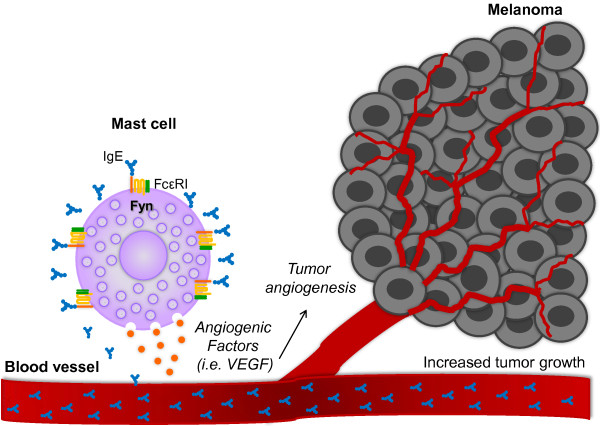
**IgE improves the pro-angiogenic properties of MC through a Fyn kinase-dependent mechanism.** Circulating IgE binds to the FcϵRI receptor on MC surface inducing the secretion of pro-angiogenic factors able to promote tumor angiogenesis and contribute to melanoma tumor growth. Fyn kinase is an important element on IgE-dependent production of pro-angiogenic factors in MC.

Our data suggest that monomeric IgE is able to potentiate the pro-tumorigenic properties of MC in a Fyn kinase-dependent fashion (Figure 8). Therefore, perturbing Fyn-activated signaling pathways to inhibit MC-dependent events leading to neovascularization of solid tumors should be evaluated in future studies.

## Methods

### Reagents

Salts, inhibitors and buffer components, as well as anti-dinitrophenyl (DNP) IgE (clone SPE-7) and DNP coupled to human serum albumin (DNP-HSA) were purchased from Sigma-Aldrich. Recombinant IL-3 was purchased from Peprotech.

### Mice

C57BL/6J (wild type: WT; stock No. 000664), mast cell-deficient B.6Cg-*Kit*^*W-sh*^ (Wsh; stock No. 005051), 129/Sv-Lyn^tm1Sor^/J (stock No. 003204) and 129-Fyn^tm1Sor^/J mice (stock No. 002271) were purchased from Jackson Laboratories. 129-Fyn^tm1Sor^/J mice were back-crossed with C57BL/6J at least five times in our animal facilities to obtain Fyn-deficient animals (Fyn −/−) on the C57BL/6J genetic background. Genotyping of each animal was performed by PCR from genomic DNA utilizing the primers and conditions suggested by the provider. Animals were maintained under pathogen-free conditions and treated in accordance with NIH guidelines. Experiments were performed with age-matched male mice of at least 8 weeks old and were approved by the animal ethics committee of Cinvestav (CICUAL, protocol 032–02 and 0478–10). When necessary, mice were euthanized by CO_2_ inhalation.

### Generation of bone marrow-derived mast cells

Bone marrow-derived mast cells (BMMCs) were generated extracting the bone marrow from both tibias of mice four to eight weeks old. Bone marrow was cultured in BMMC media (RPMI 1640 supplemented with 20 ng/mL IL-3, 0.1 mM non-essential aminoacids, 50 μM β-mercaptoethanol, 25 mM HEPES pH 7.4, 1mM pyruvate, antibiotic/antimycotic and 10% FBS) during four weeks and after that, FcϵRI receptor expression was analyzed by flow cytometry utilizing a specific positive for the IgE receptor as described [41]. To confirm functionality of BMMC cultures, routinely two million of WT or Fyn −/− BMMCs were incubated with 100 ng/ml of IgE for 20 min in 1 ml of Tyrode’s-BSA buffer (20 mM HEPES pH 7.4, 135 mM NaCl, 5 mM KCl 5 mM, 1.8 mM CaCl_2_, 1 mM MgCl_2_, 5.6 mM glucose, 0.05% bovine serum albumin; BSA) at 37°C. After incubation, different concentrations of the specific antigen (human serum albumin coupled to DNP) were added. After 30 minutes at 37°C, cell supernatants were collected and tested for β-hexosaminidase activity as described [15].

### Cell stimulation with IgE and VEGF determination

Two million BMMCs were incubated with distinct concentrations of IgE for different times at 37°C in 1 ml of BMMC media supplemented with a protease inhibitor cocktail (mini-complete, Roche). VEGF was determined in the supernatants utilizing ELISA kits from Peprotech and Invitrogen. Actinomicyn D (Act D; 5 μg/ml), Brefeldin A (BFA; 5 μg/ml), Tetanus toxin (TTx; 10 ng/ml) and PP2 (10 μM) were added to the cells 15 min previous to the stimulation [15,42] with 1000 ng/ml IgE for 8 h at 37°C.

### RNA extraction and RT-PCR

Two million BMMCs were incubated with 1000 ng/ml IgE at 37°C for different times. Total RNA was isolated utilizing Tri-Reagent and isopropanol RNA precipitation as described [43]. cDNA synthesis was performed starting with 5 μg of total RNA utilizing the Superscript First Strand System for RT-PCR from Invitrogen, following the instructions included. Reported primers were used to amplify VEGF [44] and GAPDH [45], utilizing one tenth of the cDNA reaction volume. Standardization of the number of cycles needed to obtain linear amplification of VEGF and GAPDH mRNAs in our samples was performed experimentally. Linear amplification for VEGF was obtained between 30 and 40 cycles, whereas for GAPDH it was between 25 and 30 cycles. Then, conditions for VEGF amplification were 94°C for 1.5 min; 35 cycles of 94°C for 30 sec, 58°C for 45 sec and 72°C for 45 sec and additional extension at 72°C for 10 min. Parameters for GAPDH amplification were the same but only 28 cycles of amplification were used for this gene. RT-PCR products were separated in 2% agarose gels. Photographs of each gel were quantified utilizing the LabWorks Image Acquisition and Analysis software (v 4.5) installed in a UVP image analyzer.

### Immunofluorescence

Two million BMMCs were incubated with 1000 ng/ml IgE for 8 h in 2 ml of BMMC media supplemented with a protease inhibitor cocktail (Roche) at 37°C. Cells were collected by centrifugation using a cytospin, then washed, fixed in cold acetone for 5 min at 4°C, washed again and blocked with 3% BSA, 2% goat serum and 0.01% Tween-20 in PBS for 30 min at RT. Cells were incubated with an anti-VEGF rabbit polyclonal serum (1:50; sc-507, Santa Cruz) diluted in blocking solution overnight at 4°C. After washes with PBS, slides were treated with an Alexa 568-anti-goat secondary antibody (1:500, Invitrogen) diluted in blocking solution for one hour. Finally, an incubation with 4′, 6-diamidino-2-phenylindole, dihydrochloride (DAPI, Invitrogen) for 5 min at RT was performed and slides were properly mounted. A confocal microscope (Olympus FluoView FV1000) with a 405-nm laser for DAPI and 543–nm laser for Alexa 568 was used. Fluorophores were imaged using a sequential line scan, with detection bands set at 405 to 461 nm for DAPI stain and 543 to 603 nm for Alexa 568. Each image was saved at a resolution of 512 × 512 pixel image size and analyzed. Average fluorescence intensities of VEGF per cell were measured from 13 to 33 cells per experimental group utilizing the software LabWorks 4.5.

### Western blot

Two million BMMCs were incubated with 1000 ng/ml IgE for 0, 15, 30 and 60 min in Tyrode’s buffer without BSA [16] at 37° [46]. Cell pellets were isolated and lysed on Laemmli 1X buffer. In general, fifty micrograms of total MC protein was separated in SDS-PAGE and transferred to PVDF to conduct western blot using reported solutions and methods [41]. Antibodies against phospho-pS6 ribosomal protein (Ser 235/236, 1:1000) and phospho-4E-BP1 (Thr37/46, 1:1,000) were purchased to Cell Signaling Technology. For loading control, membranes were incubated with an anti-β-actin antibody (1:10,000) from Santa Cruz. Densitometric analysis was performed utilizing an Epichemi Darkroom from UVP Systems, with the LabWorks 4.5 software.

### Standardization of IgE dose to test its effects on tumor growth

In order to evaluate the role of IgE on melanoma tumor growth, we first determined the amount of IgE that could be needed to occupy the free FcϵRI receptor on the surface of mast cells *in vivo*. C57BL/6J mice were i.v. injected with saline solution or, 25, 50, 250, 500, 750 and 1000 ng of anti-DNP IgE dissolved in 100 μL of sterile saline solution. Then, a passive anaphylaxis assay [25,26] was performed by i.v administering 100 μg DNP-HSA in 100 μl Evans blue dye (0.25%). Twenty minutes later, animals were euthanized and the limbs were removed. The amount of extravased dye in the tissue was measured as previously described [25,26]. To test the amount of free IgE after administration, C57BL/6J mice were i.v. injected with 0, 25, 50, 250, 500, 750 and 1000 ng of anti-DNP IgE and trunk blood was collected 0, 1, 7, 14, 21 and 28 days later. Each blood sample was centrifuged at 21,000 x g for 20 min at 4°C and the resultant serum was stored at −80°C. IgE levels were determined utilizing an ELISA kit (BD Biosciences).

### Reconstitution of mast cell-deficient mice

MC-deficient (Wsh) mice (eight to twelve weeks old) were reconstituted with MC as described [47]. Briefly, mice were i.v. injected with BMMCs (2×10^6^ cells) in Tyrode’s buffer without BSA and four weeks after adoptive transfer of BMMCs MC reconstitution was confirmed by histological analysis of ear pinna and passive cutaneous anaphylactic reactions. Wsh mice were reconstituted with BMMCs obtained from C57BL/6J (Wsh Rec WT) or C57BL/6J-Fyn−/− (Wsh Rec Fyn−/−) mice. Animals were inoculated with melanoma cells four to six weeks after reconstitution and tumor growth (see following section) was compared with age-matched WT and non-reconstituted animals.

### B16 melanoma cell line culture and tumor generation

B16-F1 melanoma cells obtained from ATCC (CRL-6323; low metastatic variant) were generously donated by Dr. Guadalupe Reyes, Cinvestav, and cultured as suggested by the provider. Briefly, cells were propagated in Dulbecco’s modified Eagle medium (DMEM) supplemented with 10% FBS, 100 U of penicillin and 100 μg/ml of streptomycin [23]. For tumor generation, mice were s.c inoculated into the left ear pinna with 0.5 × 10^6^ B16 melanoma cells in Tyrode’s buffer without BSA and the right ear pinna with vehicle [23]. Tumor weight development was monitored on daily basis and tumors were removed 28 days after inoculation to register weight differences between left and right ear pinna. Normal development of the tumors was routinely confirmed by histological analysis, where melanoma cells, blood vessel formation and infiltrating inflammatory cells were detected by specific dyes at different times after inoculation [48]. To test the influence of IgE on melanoma tumor growth, mice were i.v. administered with IgE (750 ng) or vehicle 24 hours previous to melanoma cell inoculation. When necessary, mice were treated with bevacizumab 10 mg/kg s.c., biweekly, starting 24 h after inoculation of melanoma cells (modified from [49]).

### Histological analysis

Biopsy specimens of the ears inoculated with vehicle or B16 melanoma cells were fixed in neutral buffered formalin (10% formaldehyde in 10 mM phosphate buffer pH 7.0) prior to paraffin embedding. Sections of 2.5 μm were cut and examined. For each treated animal (n = 2-7 per group), some ear sections were stained with hematoxylin and eosin (H&E) and other sections were stained with 0.1% toluidine blue (TB), pH 1.0 [50] in order to analyze blood vessels and mast cells, respectively. Ear pinna mast cells were counted by dividing the whole pinna area in consecutive fields length extending from the base to the tip (7.3–12.62 mm) under the 40X objective. Mast cells numbers are expressed as mean ± SEM per length of ear pinna. Images of histological sections were obtained with a microscope (Zeiss Axiostar) coupled to a digital camera (Power Shot A640). For each mouse, two sections were analyzed and counts were averaged. Blood vessel quantification was performed on 15 high-powered images of randomly selected areas of each paraffin section stained by H&E (x200). All histological measurements were done by two observers on blinded samples (control or tumor-bearing with or without IgE). In sections stained by H&E, the absolute number of large and small vessels per ear pinna was monitored by morphological analysis according to Carmeliet, 2003, *i.e.* vessels containing only the endothelial cell layer were considered small vessels, whereas those with the smooth muscle cell layer were considered to be large blood vessels [51].

### Statistical analysis

Unless specified otherwise, all data are expressed as the mean ± SEM of at least three independent experiments. Student’s t-test or Two-Way ANOVA followed by Student-Newman Keuls test was used to evaluate the significance of the differences. Statistically significant was set at P < 0.05.

## Abbreviations

MC: Mast cells; IgE: Immunoglobulin E; FcϵRI: High affinity IgE receptor; PMA: Phorbol myristate acetate; PI3K: Phosphatidylinositol-3-kinase; TNF: Tumor necrosis factor; IL-3: Interleukin-3; CCL2: Chemokine (C-C motif) ligand 2; BMMCs: Bone marrow-derived mast cells; VEGF: Vascular endothelial growth factor; S1P: Sphingosine 1 phosphate; DNP: Anti-dinitrophenol; DNP-HSA: DNP coupled to human serum albumin; Act D: Actinomicyn D; BFA: Brefeldin A; TTx: Tetanus Toxin; DAPI: 4′, 6-diamidino-2-phenylindole, dihydrochloride; S6: Ribosomal protein S6; 4E-BP1: Eukaryotic initiation factor 4E binding protein 1; WT: Wild type; Fyn −/−: Fyn-deficient; Wsh: Mast cell-deficient B.6Cg-KitW-sh mice; i.v.: Intravenously; s.c: Subcutaneously; DMEM: Dulbecco’s modified Eagle medium.

## Competing interests

The authors declare that they have no competing interests.

## Authors’ contributions

GYJ-A contributed to the design of the experiments, carried out immunofluorescence, performed the tumor studies, participated in the *in vitro* studies, contributed to the interpretation of the results, participated in the statistical analysis of the study and drafted the manuscript. AI-S generated BMMCs, carried out passive cutaneous anaphylaxis studies and Western blots, determined VEGF mRNA quantification and VEGF secretion. DG performed blood vessels quantification. ML contributed to the design of the experiments, provided vital subjects for research and edited manuscript. CG-E conceived the study, designed the research, coordinate the activities of each contributor, provided vital subjects for research and edited manuscript. All authors read and approved the final manuscript.
